# Non-Hodgkin's Lymphoma of the Middle Ear Presenting as Mastoiditis

**DOI:** 10.1155/2018/7639784

**Published:** 2018-10-17

**Authors:** Marwan Alqunaee, Abdullah Aldaihani, Mohammed AlHajery

**Affiliations:** ^1^Otorhinolaryngology, Head and Neck Surgery Resident, Kuwait Institute of Medical Specialization, Kuwait City, Kuwait; ^2^Department of Surgery, Adan Hospital, Hadiya, Kuwait; ^3^Department of ENT, Zain and Al Sabah Hospitals, Kuwait City, Kuwait

## Abstract

Lymphoma originating from the middle ear is rare. The diagnosis of lymphoma as with other cancers of the temporal bone is often made late, and this has a negative implication on the treatment and prognosis of the condition. The delay of diagnosis is mainly due to the similar presentation shared with other benign conditions of the middle ear. We present a case of a 62-year-old man who was treated as a case of chronic otitis media for a period of time before presenting with advanced symptoms; a final diagnosis of lymphoma of the middle ear was given. Other similar cases in the literature are discussed and reviewed. Severe and persistent symptoms of the middle ear should raise red flags and warrant detailed investigations.

## 1. Introduction

Lymphoma is a cancer of the reticuloendothelial system; it has two subtypes, Hodgkin's lymphoma and non-Hodgkin's lymphoma. Furthermore, lymphomas constitute 10% of all head and neck tumors; 60% of these are non-Hodgkin's lymphoma and the other 40% are Hodgkin's lymphoma.

Diffuse large cell B lymphoma is the largest subunit of non-Hodgkin's lymphoma, and it is characterized by extranodal involvement. The most common sites of extranodal involvement are the stomach and the gastrointestinal tract; however, lymphoma can present in any type of tissues. The bony involvement comprises 1-2% of malignant lymphomas and 3–5% of extranodal lymphomas; involvement of temporal bone is extremely rare with only few reported cases in the literature [1–3].

## 2. Case

A 62-year-old man presented to the otolaryngology clinic with a House-Brackmann grade 4 left lower motor facial nerve palsy with a 10-day history of left postauricular pain. This was preceded by 6 months of intermittent purulent discharge from the left ear for which he received multiple antibiotic courses. On examination, the patient was vitally stable; however, he was febrile. The left postauricular area was mildly tender on palpation and the overlying skin was normal. Otoscopic examination of the left ear was only significant for an erythematous and retracted tympanic membrane. The rest of the examination of the right ear along with a full head and neck exam was unremarkable. White blood count was elevated with 13900 (76% neutrophils, 16.2% lymphocytes, and 8.3% monocytes).

**Figure 1:**
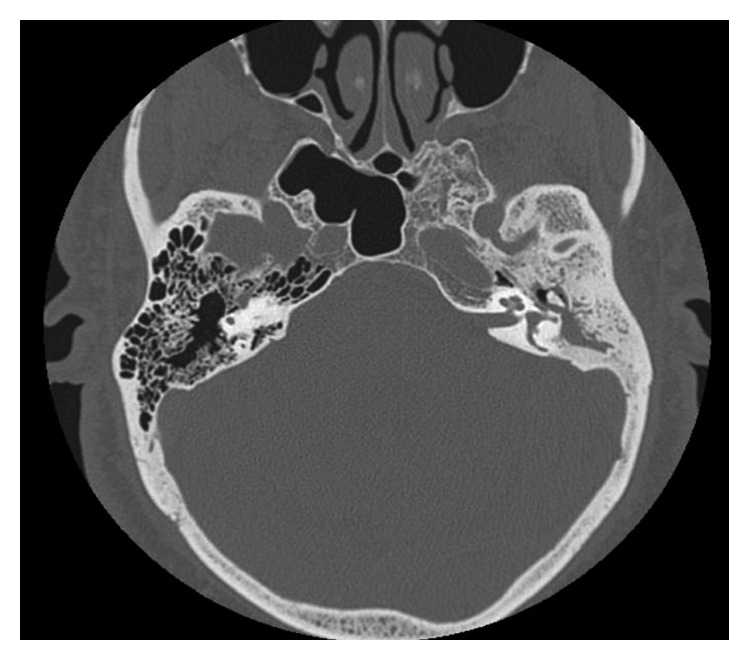
High-resolution CT scan (axial cut) of the temporal bone. Total opacification of the left-sided hypo- and mesotympanum by a soft tissue density. Mastoid air cells totally opacified with resorption of septae and partially sclerosed bone. Partial dehiscence of the mastoid portion of the facial nerve canal. The left tympanic membrane is thickened and retracted inwards. The left middle ear ossicles and scutum are intact. Normal aeration of the external auditory canals with intact bony and cartilaginous boundaries. The right middle ear cavity is normally aerated with intact ossicles. The right mastoid air cells are unremarkable.

**Figure 2:**
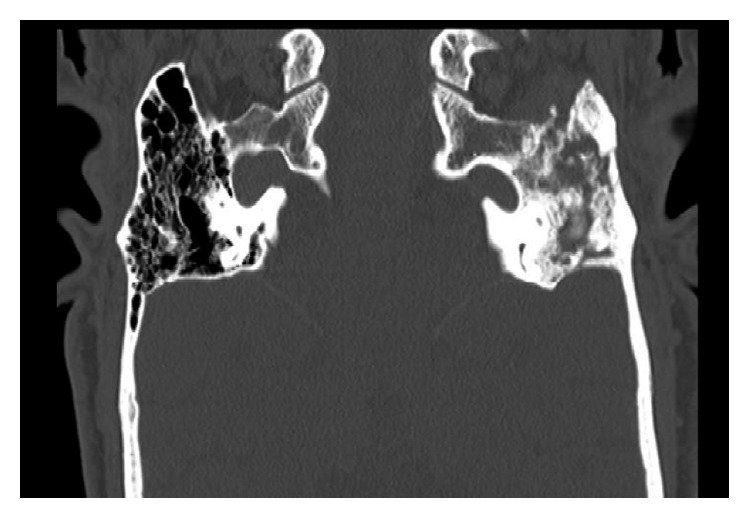
High-resolution CT scan (coronal cut) of the temporal bone. Total opacification of the left-sided hypo- and mesotympanum by a soft tissue density. Mastoid air cells totally opacified with resorption of septae and partially sclerosed bone. Partial dehiscence of the mastoid portion of the facial nerve canal. The left tympanic membrane is thickened and retracted inwards. The left middle ear ossicles and scutum are intact. Normal aeration of the external auditory canals with intact bony and cartilaginous boundaries. The right middle ear cavity is normally aerated with intact ossicles. The right mastoid air cells are unremarkable.

**Figure 3:**
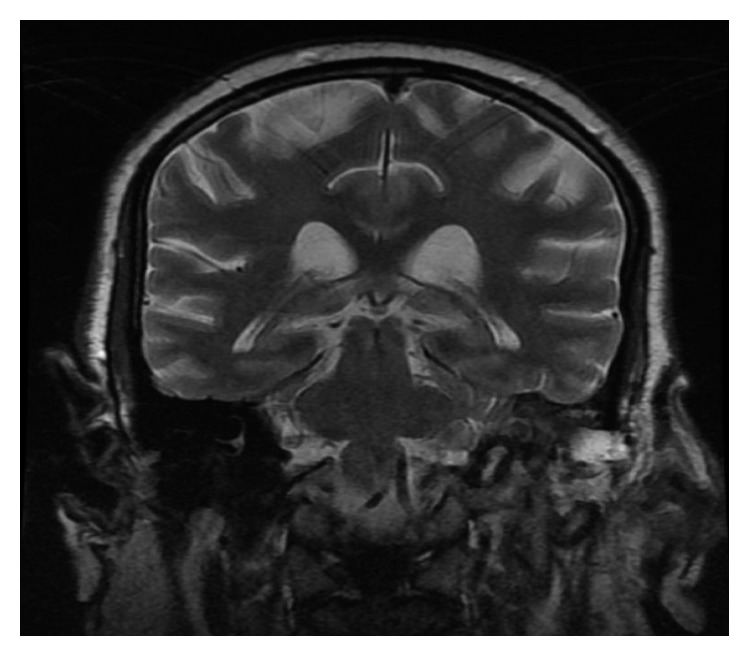
MRI of cerebellopontine angles and posterior fossa (coronal cut). Opacification of the left mastoid air cells and middle ear cavity with soft tissue signal which elicits low signal in T1WI and high signal in T2WI with restricted diffusion in DWI. There is homogeneous enhancement following IV contrast administration. No evidence of adjacent intracranial abscess formation or sinus thrombosis. The region of brain stem and cerebellar hemispheres are normal. No focal lesions are seen. Both the VII and VIII cranial nerves show normal course and signal intensity. No intra- or extra-canalicular enhancing lesion is seen.

**Figure 4:**
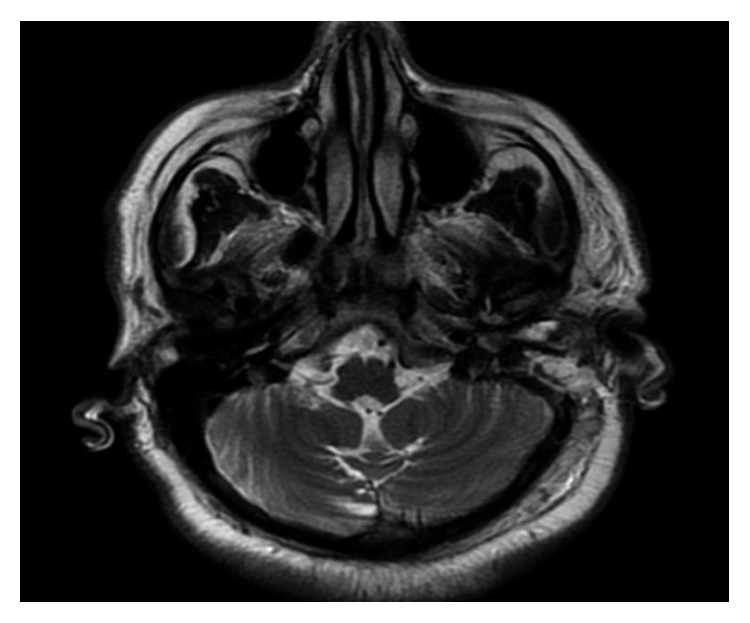
MRI of cerebellopontine angles and posterior fossa (axial cut). Opacification of the left mastoid air cells and middle ear cavity with soft tissue signal which elicits low signal in T1WI and high signal in T2WI with restricted diffusion in DWI. There is homogeneous enhancement following IV contrast administration. No evidence of adjacent intracranial abscess formation or sinus thrombosis. The region of brain stem and cerebellar hemispheres are normal. No focal lesions are seen. Both the VII and VIII cranial nerves show normal course and signal intensity. No intra- or extra-canalicular enhancing lesion is seen.

The patient was admitted to the hospital and had an initial diagnosis of mastoiditis with facial nerve paralysis. He was started on intravenous antibiotics. High-resolution computed tomography (HRCT) and magnetic resonance imaging were performed (Figures 1–4).

Due to the severe complication of the facial nerve palsy a decision was made for surgical intervention; cortical mastoidectomy with facial nerve decompression and left middle ear exploration was performed. Granulation tissue in the mastoid air cells and the middle ear were encountered and removed. Biopsies were also taken and sent for histopathology.

**Figure 5:**
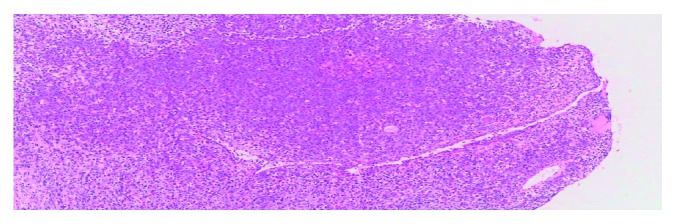
Microphotograph shows diffuse infiltration of large neoplastic lymphoid cells. HEx100x.

The histopathological assessment of the tissue revealed diffuse proliferation of large monomorphic atypical lymphoid cells admixed with few medium-sized cells (centroblastic and prominent immunoblastic lymphoid cells) (Figure 5).

The cells showed scant to moderate amphophilic cytoplasm, vesicular nuclei, or focal irregular chromatin clumping with prominent 1-2 nucleoli.

**Figure 6:**
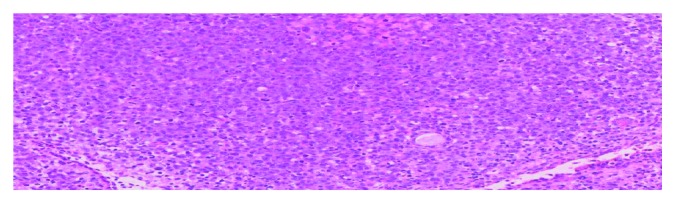
Microphotograph shows atypical large lymphoid cells with prominent 1-2 nucleoli, subendothelial infiltration, frequent mitoses, and a pseudorosette. HEx200x.

**Figure 7:**
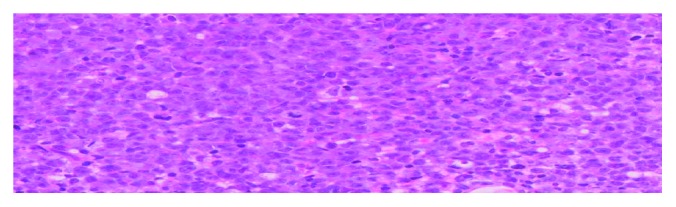
High-power view of transformed lymphoid cells and mitoses. HEx400x.

Frequent mitoses and few tumor giant cells were regarded along with subendothelial infiltrate and occasional pseudorosette (Figures 6 and 7).

**Figure 8:**
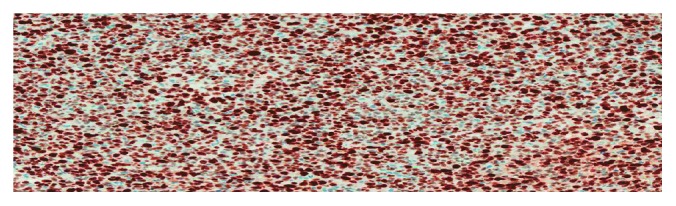
Ki67 by immunohistochemistry shows high proliferation index (80–90%). 200x.

The proliferative fraction as detected by Ki67 immunostaining is very high (80–90% positivity) (Figure 8).

The patient was then transferred to the oncology unit for staging and further management. There was no distant spread and the patient was treated with courses of chemotherapy. At the most recent follow-up his ear symptoms of otalgia and persistent discharge have resolved; however, he still showed a House-Brackmann grade 3 left lower motor neuron facial palsy.

## 3. Discussion

Carcinoma of the temporal bone is rare, and most of the pathological diagnosis in such cases turns out to be squamous cell carcinoma. In addition, adenoid cystic carcinoma, adenocarcinoma, and basal cell carcinomas along with metastasis are also reported. Other malignant tumors that can occur in the temporal bone are melanoma, rhabdomyosarcoma, chondrosarcoma, and lymphoma [4].

Lymphoma of the middle ear can arise from the mucosa of the mastoid antrum, tympanum, or the tympanic orifice of the eustachian tube. In addition to that it can arise primarily from the temporal bone, as in our case, which is in the mastoid part of the temporal bone.

Diagnosis of cancer in the middle ear and the mastoid process is seldom made early; this can contribute to the unique anatomy of middle ear and the fact that many benign pathologies such as otitis media and its complications share similar clinical presentations; most commonly reported symptoms include otorrhea, otalgia, aural fullness, tinnitus, and hearing loss [5].

Severe hearing loss, facial nerve weakness, and vestibular dysfunction are advanced sequel of middle ear pathology that should raise red flags for a more sinister cause. In addition to malignancy, autoimmune conditions such as granulomatosis with polyangiitis and sarcoidosis and immunodeficiency conditions should also be considered in the differential diagnosis of cases of refractory chronic otitis media with facial nerve palsy. Delay in management of such cases usually occurs due to a low index of suspicion of the treating physician.

Saito et al. presented a similar case in a 58-year-old woman who presented with headache and auditory disturbances in addition to otitis media; further investigations revealed that the patient has transverse sinus thrombosis. Surgery was performed 6 years after initial presentation, when only the diagnosis of lymphoma was made. The patient then recovered fully after a course of chemotherapy. They have also reviewed the literature and found 10 cases of lymphoma arising around the middle ear; the most common presentations were facial nerve palsy and otorrhea which is consistent with the presentation of our case [6].

Roberts et al. have presented a case of a 78-year-old woman with large B-cell lymphoma of the middle ear presenting as chronic otomastoiditis. However, in their case there was no involvement of any of the cranial nerves, and emphasis was made on the early detection of temporal bone malignancy through neurotological examination and imaging as early diagnosis and aggressive chemotherapeutic treatment for primary lymphoma of the middle ear lymphoma are crucial for a good outcome [7].

Our case presented similarly to other cases in the literature; however, earlier detection within the period where he had persistent symptoms refractory to medical treatment may have prevented the advanced complication of facial nerve palsy and increased his chances of cure.

## 4. Conclusion

Lymphoma in the temporal bone is very rare; it may manifest with symptoms that are similar to other common benign middle ear pathologies. In severe and persistent symptoms a malignant cause should be put into consideration; this ensures early detection and thus management of the condition.
